# Editorial Comment: Role of pelvicalyceal anatomy in the outcomes of retrograde intrarenal surgery (RIRS) for lower pole stones: outcomes with a systematic review of literature

**DOI:** 10.1590/S1677-5538.IBJU.2020.02.05

**Published:** 2020-01-10

**Authors:** Alexandre Danilovic

**Affiliations:** 1 Hospital das Clínicas Faculdade de Medicina USP São PauloSP Brasil Serviço de Urologia, Hospital das Clínicas da Faculdade de Medicina da USP - HCFMUSP, São Paulo , SP , Brasil

Karim SS ^1^ ,Hanna L ^1^ ,Geraghty R ^1^ ,Somani BK ^2^

^1^ Department of Urology, University Hospital Southampton NHS Foundation Trust, Southampton, SO16 6YD, UK; ^2^ Department of Urology, University Hospital Southampton NHS Foundation Trust, Southampton, SO16 6YD, UK

Urolithiasis. 2019;1. [Epub ahead of print]

**DOI:** 10.1007/s00240-019-01150-0 | **ACCESS:** 10.1007/s00240-019-01150-0

## COMMENT

During the last decade, retrograde intrarenal surgery (RIRS) has been widely used to treat most kidney stones. Sometimes, indications of RIRS exceed the stone size limit of the guidelines and often neglect the collecting system anatomy. RIRS is currently recommended for treatment of kidney stones up to 20 mm and there is no established parameter of the collecting system for the indication of RIRS in the guidelines ( 1 ).

In this study, the authors reviewed previously published papers and themselves investigated the role of pelvicalyceal anatomy in the outcomes of RIRS for lower pole stones. They found steep infundibular pelvic angle (IPA) less than 30°, operative time and larger stone size were significant predictors of residual stone fragments. Moreover, IPA was the most important predictor for being stone free in the lower pole.

Other authors also demonstrated a critical role of IPA for outcomes of RIRS. Using computerized tomography (CT), they demonstrated a steep IPA less than 41° was a predictor for residual fragments after RIRS ( 2 ). Therefore, it is crucial to evaluate IPA in the preoperative CT to better predict the stone free rate of RIRS up to 20 mm.
